# Prediction of Treatment Response in Patients with Major Depressive Disorder: A Meta-Analysis of Functional Magnetic Resonance Imaging Studies

**DOI:** 10.1192/j.eurpsy.2022.758

**Published:** 2022-09-01

**Authors:** M. Torres, P. Manghera, C. Miller

**Affiliations:** 1 California State University, Fresno, Psychology, Clovis, United States of America; 2 California State University, Fresno, Psychology, Fresno, United States of America

**Keywords:** treatment response, meta-analysis, Functional Magnetic Resonance Imaging, major depressive disorder

## Abstract

**Introduction:**

Identifying the optimal treatment for individuals with major depressive disorder (MDD) is often a long and complicated process. Functional magnetic resonance imaging (fMRI) studies have been used to help predict and explain differences in treatment response among individuals with MDD.

**Objectives:**

We conducted a comprehensive meta-analysis of treatment prediction studies utilizing fMRI in patients with MDD to provide evidence that neural activity can be used to predict response to antidepressant treatment.

**Methods:**

A multi-level kernel density analysis was applied to these primary fMRI studies, in which we analyzed brain activation patterns of depressed patients (N= 364) before receiving antidepressant treatment.

**Results:**

The results of this analysis demonstrated that hyperactivity in six brain regions significantly predicted treatment response in patients with MDD: the right anterior cingulate, right cuneus, left fusiform gyrus, left middle frontal gyrus, right cingulate gyrus, and left superior frontal gyrus.

**Conclusions:**

This study provides evidence that neural activity, as measured by standard fMRI paradigms, can be used to successfully predict response to antidepressant treatment. This may be used in the future clinically to improve decision-making processes and treatment outcomes for patients.

**Disclosure:**

No significant relationships.

